# Maternal diet induces persistent DNA methylation changes in the muscle of beef calves

**DOI:** 10.1038/s41598-023-28896-3

**Published:** 2023-01-28

**Authors:** Rocío Amorín, Lihe Liu, Philipe Moriel, Nicolás DiLorenzo, Phillip A. Lancaster, Francisco Peñagaricano

**Affiliations:** 1 University of Florida Genetics Institute, University of Florida, Gainesville, FL 32611 USA; 2 Department of Animal and Dairy Sciences, University of Wisconsin-Madison, 1675 Observatory Dr., Madison, WI 53706 USA; 3 Range Cattle Research and Education Center, University of Florida, Ona, FL 33865 USA; 4 North Florida Research and Education Center, University of Florida, Marianna, FL 32351 USA; 5 Department of Clinical Sciences, Kansas State University, Manhattan, KS 66506 USA

**Keywords:** Agricultural genetics, Animal breeding, Epigenomics, Genomics

## Abstract

Maternal nutrition during pregnancy can induce epigenetic alterations in the fetal genome, such as changes in DNA methylation. It remains unclear whether these epigenetic alterations due to changes in maternal nutrition are transitory or persist over time. Here, we hypothesized that maternal methionine supplementation during preconception and early pregnancy could alter the fetal epigenome, and some of these alterations could persist throughout different developmental stages of the offspring. Beef cows were randomly assigned to either a control or a methionine-rich diet from − 30 to + 90 d, relative to the beginning of the breeding season. The methylome of loin muscle from the same bull calves (n = 10 per maternal diet) at 30 and 200 days of age were evaluated using whole-genome bisulfite sequencing. Notably, a total of 28,310 cytosines showed persistent methylation differences over time between maternal diets (q-value < 0.10, methylation change > 20%). These differentially methylated cytosines were in the transcription start sites, exons, or splice sites of 341 annotated genes. Over-representation analysis revealed that these differentially methylated genes are involved in muscle contraction, DNA and histone methylation, mitochondrial function, reactive oxygen species homeostasis, autophagy, and PI3K signaling pathway, among other functions. In addition, some of the persistently, differentially methylated cytosines were found in CpG islands upstream of genes implicated in mitochondrial activities and immune response. Overall, our study provides evidence that a maternal methionine-rich diet altered fetal epigenome, and some of these epigenetic changes persisted over time.

## Introduction

Fetal programming is defined as the fetal response to an intrauterine stimulus or insult during a critical developmental time window, resulting in an alteration of the developmental path. This phenomenon refers to adaptations that occur during fetal life in response to diverse stimuli, such as changes in nutrition, heat stress or exposure to diseases, among others. These adjustments occur with the idea that the post-natal environment will coincide with the prenatal (in utero) environment^1^. In many cases, as shown in humans and animal models, fetal programming has negative consequences altering postnatal growth and body composition, causing metabolic disorders and dysfunction of various organs^2^. However, in livestock, this phenomenon has also been associated with positive changes in the performance of the offspring. For instance, supplementing ewes with linoleic acid during late gestation led to an increase in the survival of lambs at parturition during cold stress^3^. Furthermore, protein supplementation in beef cattle during late gestation, resulted in increased body weight and fertility in heifers^4^, as well as increased fat deposition in steers^5^. These results are relevant for food livestock production as improving traits such as postnatal growth, body composition, and carcass weight, may improve beef quality, resulting in economic benefits.

The mechanisms underlaying fetal programming are not well understood, however they are thought to be both physiological and epigenetic, with DNA methylation changes playing an important role^6^. DNA methylation is an epigenetic modification comprised of the addition of a methyl group on the C5 position of the cytosine ring, an alteration that is particularly important due to its mitotic heritability. This modification happens for the most part in CpG dinucleotides, although it can be found in other contexts, such as CHG and CHH where H can be adenosines, cytosines or thymines. While CpG dinucleotides are depleted throughout the genome, they are found in high concentrations in CpG islands^7^. Roughly 70% of annotated gene promoters overlap a CpG island^8^, which holds importance when considering that gene expression is altered through changes in the methylation status of the promoter^9,10^. Changes in DNA methylation may prompt phenotypic changes that can be permanent and transgenerational^11^.

Maternal nutrition can particularly influence DNA methylation through the one carbon cycle, a metabolic pathway dependent on micronutrients, such as folate, vitamin B6, vitamin B12, and methionine. Through a chain of reactions, methionine is converted to S-adenosylmethionine, which acts as a universal methyl donor^12^. Our team has shown that changes in methionine supplementation around the time of conception in beef cattle causes epigenetic modifications in the offspring. Indeed, we have identified significant changes in the muscle epigenome of 30-day-old beef calves due to maternal methionine supplementation, including changes in gene coexpression patterns^13^ and alternative splicing^14^. Remarkably, some of these transcriptomic changes were mediated by changes in DNA methylation. However, it remains unclear whether these DNA methylation changes are transitory or persist over time. As such, in this study we decided to evaluate the muscle epigenome of the same group of beef calves that we had previously evaluated at 30 days of age, but this time at 200 days of age. We hypothesized that maternal methionine supplementation during preconception and early pregnancy would alter the fetal epigenome, and some of these alterations would persist throughout different developmental stages of the offspring.

## Materials and methods

### Ethics statement

All the animal procedures were approved by the University of Florida Institutional Animal Care and Use Committee (IACUC #2014408583) of the University of Florida. The experiment was conducted at the UF/IFAS Range Cattle Research and Education Center (Ona, FL) and was performed in accordance with relevant guidelines and regulations. Reporting in this manuscript follows the recommendations of the ARRIVE guidelines (https://arriveguidelines.org).

### Animals and experimental design

Eighty Brangus-Angus cows were randomly assigned to one of two nutritional treatments during days − 30 to 90, relative to the beginning of the breeding season^15^. These treatments consisted of a control diet based on limpograss hay supplemented with molasses and urea; and a methionine-rich diet consisting of the control diet added with 10 g of MetaSmart Liquid (Adisseo, Alpharetta, GA) providing 3.7 g of rumen-protected methionine per head per day. *Longissimus dorsi* muscle samples were collected from the same 20 calves (10 calves per maternal dietary treatment) at 30 and 200 days of age. Approximately 50 mg of muscle samples were collected from the longissimus dorsi muscle located above the 11th and 12th rib using a Tru-Cut biopsy device. Immediately after sample collection, muscle samples were snap-frozen with liquid nitrogen. Muscle samples were stored at − 80 °C until DNA extraction.

### DNA extraction, library preparation and sequencing

Total DNA was extracted from muscle samples for whole-genome bisulfite sequencing analysis. Extraction, library construction, bisulfite treatment and sequencing were performed by Novogene Bioinformatics Technology Co., Ltd (Beijing, China). After extraction, DNA quality and quantity were tested using Nanodrop (OD260/OD280) and Qubit^®^ 2.0. The DNA was then fragmented into 200-300 bp fragments using Covaris S220. Terminal repairing, A-ligation and adapters ligation were performed to the DNA fragments. The resulting DNA library was treated with bisulfite using EZ DNA Methylation Gold Kit from Zymo Research, and then subjected to size selection and PCR amplification. Library concentration was firstly quantified by Qubit^®^ 2.0, and then was diluted to 1 ng/ul before checking insert size on Agilent 2100. Finally, libraries were sequenced with Illumina’s HiSeq 3000 using 150-bp paired-end reads. Whole-genome bisulfite sequencing data can be accessed by GEO with the accession number GSE117194 and GSE218011.

### Bisulfite-seq quality control and editing

The quality of the sequencing reads was evaluated using the software FastQC (version 0.11.7, Babraham Bioinformatics, UK). Adaptor removal and trimming was performed using the software Trim Galore (version 0.4.4, Babraham Bioinformatics, UK) with the following parameters: --clip_R1 10 --clip_R2 10 --three_prime_clip_R1 20 --three_prime_clip_R2 20.

### Bisulfite-seq mapping

After quality control and editing, the resulting paired-end sequencing reads were aligned to the bovine reference genome ARS-UCD1.2 using the software Bismark (version 0.17.0, Babraham Bioinformatics, UK)^16^. Duplicated read alignments were detected and removed using the Bismark tool *deduplicate_bismark*. Finally, Bismark tool *methylation extractor* was used for methylation calling using the following parameters: --paired-end, --comprehensive, --bedGraph, and --cytosine_report.

### Differentially methylated cytosines

Differential methylated cytosines between maternal diets in each time point were identified using a logistic regressions implemented in the *R* package Methylkit (version 1.0.0)^17^. Only cytosines with read coverage ≥ 8 in a CpG context were evaluated. Differentially methylated cytosines were defined as those having methylation percentage changes between maternal treatments ≥ 20% and q-values ≤ 0.10.

### Mapping cytosines to genomic features

Both differentially methylated (significant) and non-differentially methylated (non-significant) cytosines were mapped to different genomic features, including coding and non-coding regions, using the *R* package rtracklayer (version 1.50.0)^18^. Specifically, cytosines were assigned to (i) promoter regions, defined as 4.8 kb upstream to the transcription start sites, (ii) transcription start sites, defined as 200 bp upstream to the first exon, (iii) coding and non-coding exons, (iv) splice sites, including 5’ donor and 3’ acceptor sites, defined as the first 50 bp and the last 50 bp of an intron, respectively, (v) intronic regions (excluding splice sites), and finally (vi) intergenic regions.

### Over-representation analysis

Functional terms from different databases, including Gene Ontology (GO)^19^, Kyoto Encyclopedia of Genes and Genomes (KEGG)^20^, InterPro^21^, Reactome^22^, Medical Subject Headings (MeSH)^23^ and Molecular Signatures Database (MSigDB)^24^, were interrogated using a Fisher’s exact test. Only genes with at least one cytosine evaluated in both time points located in either the transcription start site, 5’ donor site, 3’ acceptor site, or exons, were considered in this analysis. Significant genes were defined as those genes having at least one cytosine that was persistently differentially methylated in either the transcription start site, 5’ donor site, 3’ acceptor site, or exons. All these analyses were performed using the R package EnrichKit (https://github.com/liulihe954/EnrichKit). Note that that the functional terms evaluated are not independent, i.e., gene-set databases have a hierarchical structure, and hence, a classical multiple testing approach would be overly conservative, and it was not performed.

### CpG islands

CpG islands were identified with the software GenomeCluster (version 1.0)^25^. These CpG islands were then associated to differentially methylated cytosines and genes based on chromosome coordinates.

## Results

### Bisulfite sequencing of muscle samples

Total DNA was successfully extracted, processed, and sequenced from a total of 16 samples at 30 days of age and 19 samples at 200 days of age. Roughly 330 M paired-end reads per muscle sample were generated using whole-genome bisulfite sequencing. Reads were mapped to the bovine reference genome ARS-UCD1.2 using the software *Bismark* yielding a 72% mapping rate. Supplementary Information 1 shows the results of the alignment of the sequencing reads to the bovine reference genome.

### Identification of persistent DNA methylation changes

**Figure 1:**
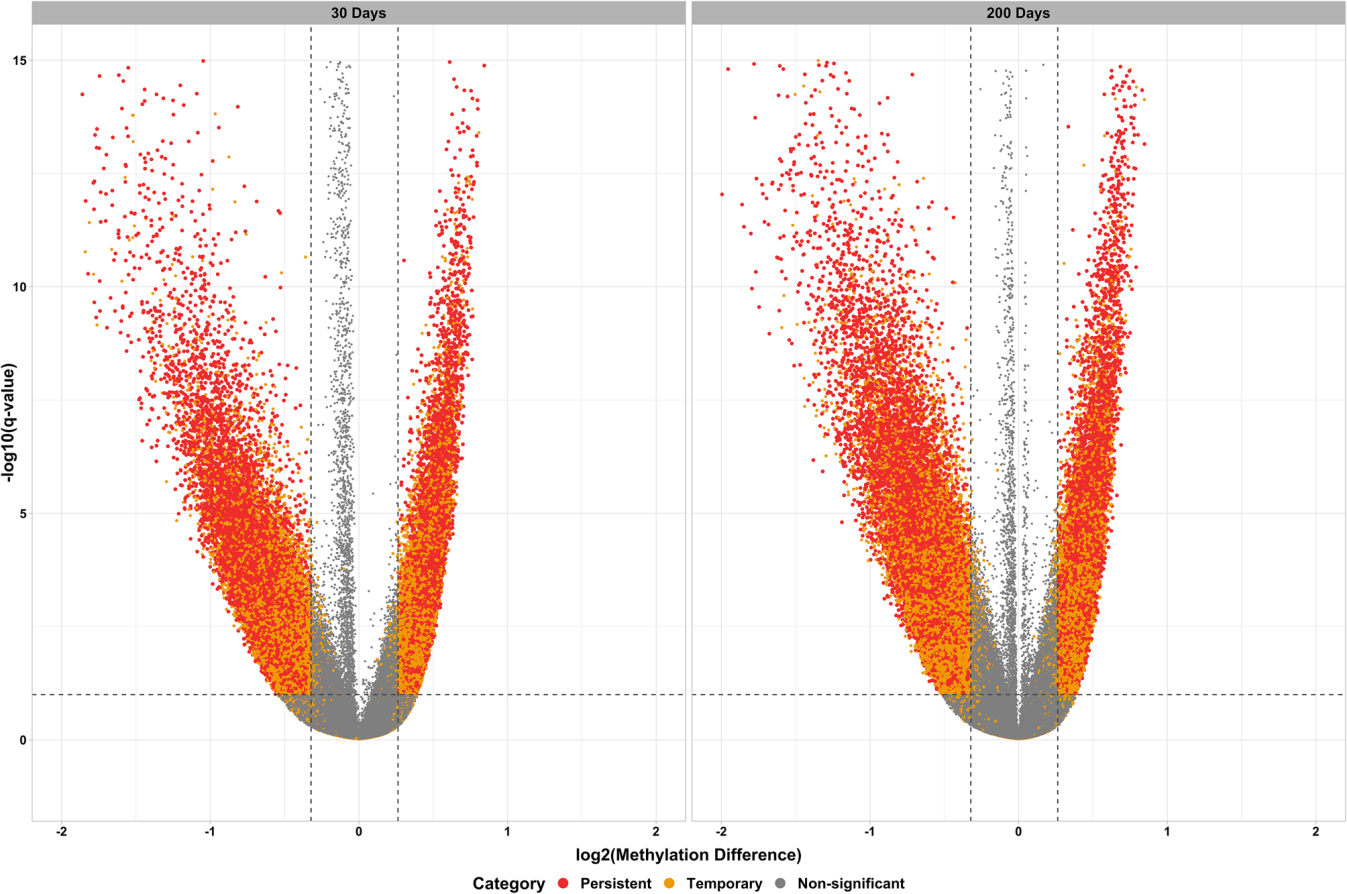
Differential methylation analysis. Plots of statistical significance (-log10 q-value) versus magnitude of change (log2 methylation difference) at 30 and 200 days of age.

**Figure 2:**
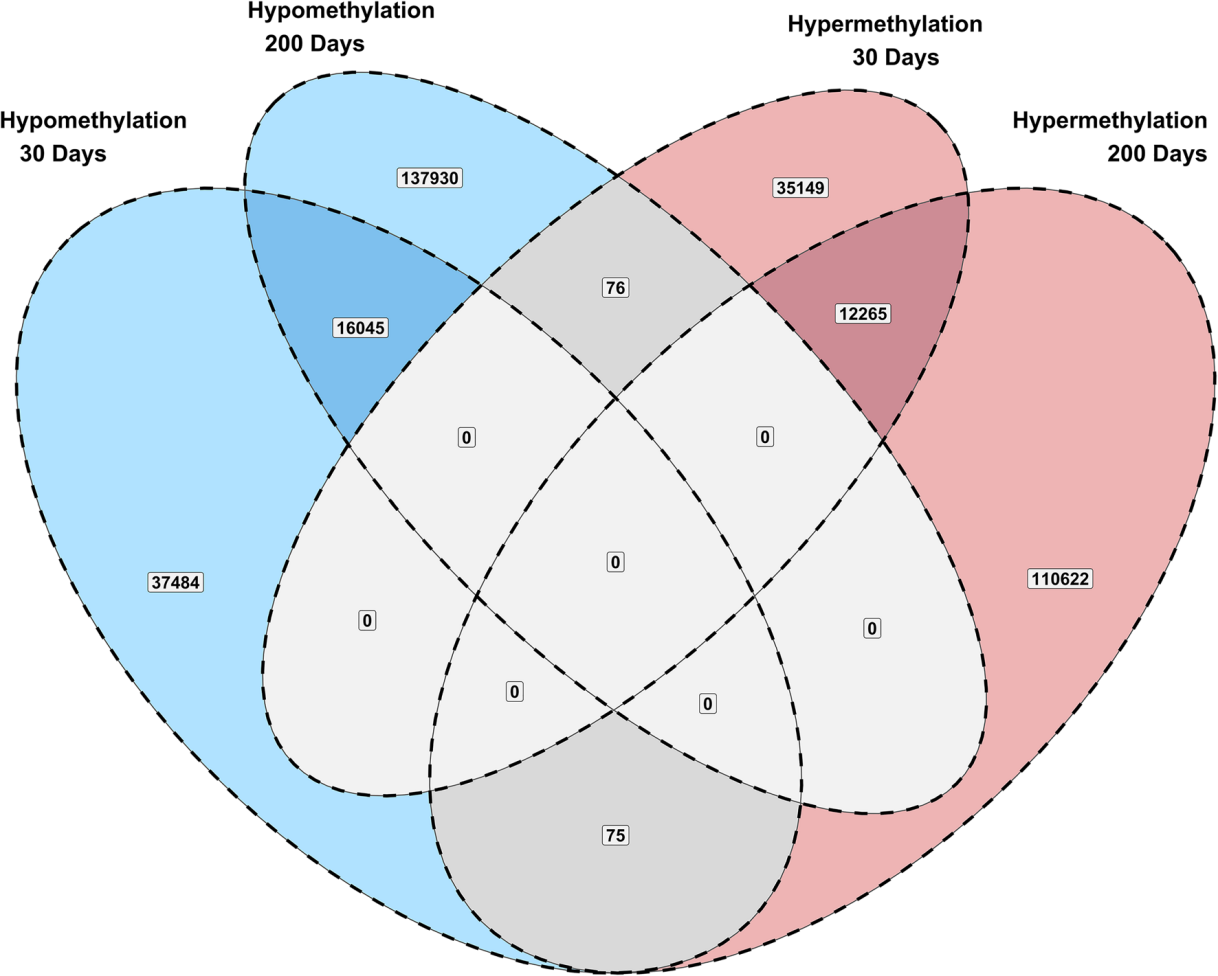
Summary of persistent DNA methylation changes over time.

Changes in DNA methylation due to maternal nutrition were evaluated at each time point separately. Differentially methylated cytosines were identified using the following criteria: read coverage ≥ 8, methylation change ≥ 20%, and q-value ≤ 0.10. Figure 1 shows the results of the differential methylation analysis as statistical significance versus magnitude of change. Notably, a total of 28,310 cytosines showed persistent methylation differences over time. Figure 2 shows the number of cytosines that showed persistent hypo- and hyper-methylation differences. Out of 28,310 cytosines that showed persistent methylation differences, 12,265 were hypermethylated and 16,045 were hypomethylated due to the maternal methionine-rich diet.

### Localization of persistent DNA methylation changes

**Figure 3:**
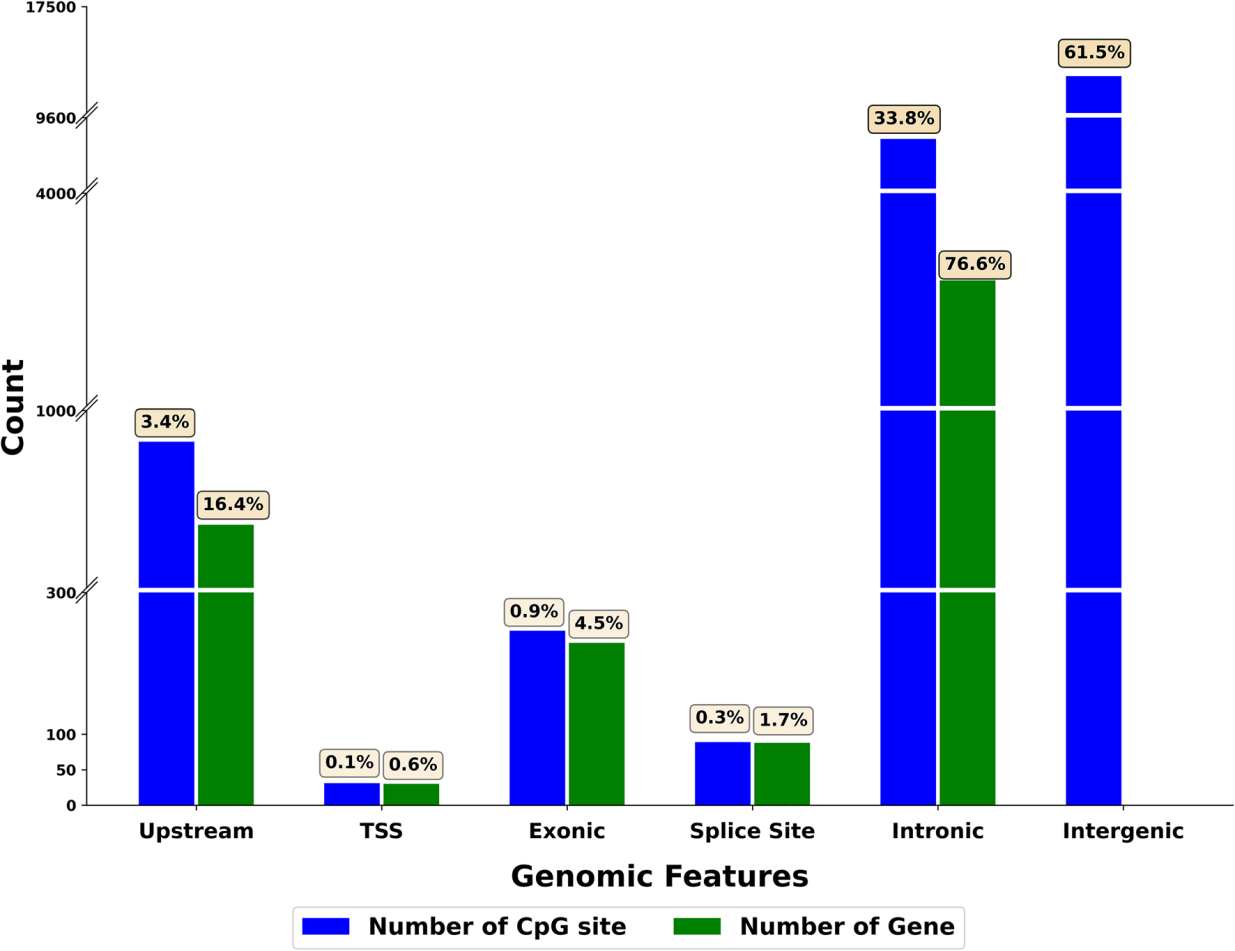
Genomic localization of persistent DNA methylation changes. This figure was created with R software (R version 4.0.3, https://www.r-project.org).

Using the ARS-UCD1.2 genome annotation, the cytosines that showed persistent methylation changes across time were categorized as (i) located in promoter regions, (ii) located in transcription start sites, (iii) located within exons, (iv) located within splice sites, both 5’ donor or 3’ acceptor sites, (v) located within introns (excluding the splice sites), or (vi) located in an intergenic region. Figure 3 shows the genomic localization of the cytosines that showed persistent DNA methylation changes across time. The majority of these significant cytosines (n = 17,400) were in intergenic regions. The remaining significant cytosines were located within or very near annotated genes, namely introns (n = 9582), upstream (n = 959), exons (n = 248), splice sites (n = 89), and transcription start sites (n = 32). Supplementary Information 2 reports the full list of differentially methylated cytosines and the corresponding genomic regions.

### Functional characterization of persistent DNA methylation changes

**Figure 4:**
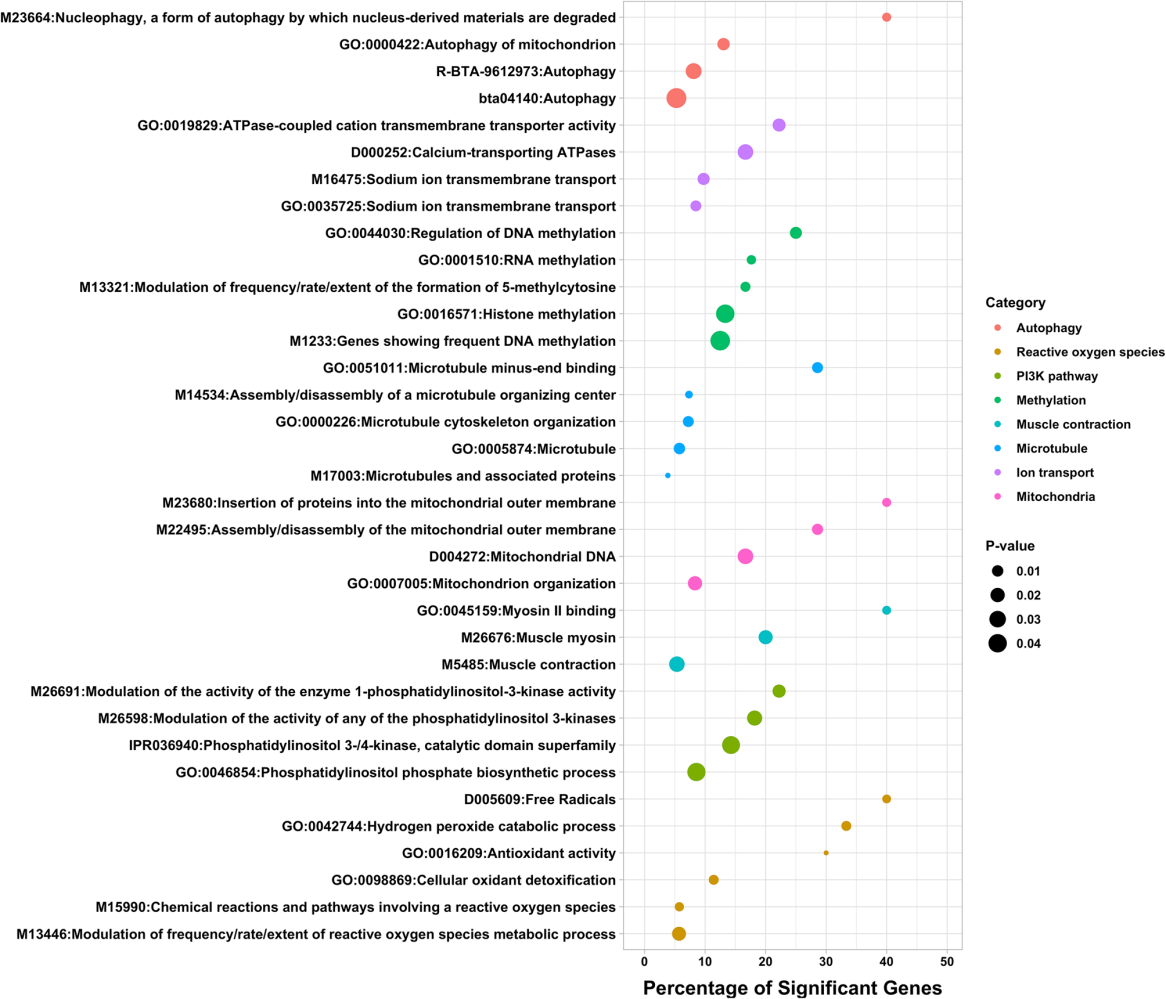
Functional characterization of persistent DNA methylation changes. The y-axis displays the names of the functional terms, the size of the dots represents the significance of the enrichment (-log10 *P*-Value, Fisher’s exact test) and x-axis represents the percentage of significant genes in each gene-set.

A total of 341 genes showed persistent DNA methylation changes in important genomic features, including transcription start sites, exons, and splice sites. An over-representation analysis, also known as gene-set analysis, was performed to gain more insight into the functional roles of these genes with persistent DNA methylation changes, and further characterize the biological processes and molecular mechanisms that could be disturbed by maternal methionine supplementation. Figure 4 shows a list of terms that were significantly enriched with genes that harbored cytosines with persistent methylation changes. Several functions and processes were revealed, including terms related to muscle contraction, ion transport, DNA and histone methylation, mitochondrial function, reactive oxygen species homeostasis, autophagy, and PI3K signaling pathway, among other functions. Supplementary Information 3 shows the full list of significant functional terms, including term ID, term name, total number of genes, number of significant genes, percentage of significant genes, and Fisher’s *P* value.

### Detection of CpG islands with persistent DNA methylation changes

Methylation in DNA happens mostly at CpG sites, and these seem to be highly concentrated in CpG islands. Note that CpG islands have been associated to regulatory regions of the DNA, therefore a methylation analysis of these locations is of relevance. Notably, a total of 10 CpG islands harbored cytosines with persistent methylation changes across time. These 10 CpG islands were located either in promoter regions or transcription start sites of 10 different bovine genes, including *SKINT1* and *ZAP70*, which are implicated in the immune response, *SGO1* which is involved in the mitotic process, and *COX17* which is involved in mitochondrial complex IV assembly. Supplementary Information 4 shows the full list of relevant CpG islands, including the location of the significant cytosines, and the corresponding gene IDs.

## Discussion

Previous studies have shown that different intrauterine insults have permanent and sometimes transgenerational consequences in the physiology and health of the offspring. The present study was designed to assess whether maternal methionine supplementation could alter the fetal epigenome and, more importantly, whether some of these alterations might persist over time. Indeed, maternal methionine supplementation significantly impacted the epigenome of the offspring, both at 30 and 200 days of age. It is well-documented that intrauterine insults, such as maternal nutrition, can alter epigenetic marks of the fetal genome, such as DNA methylation^26^, and our findings provide additional evidence. More importantly, some of the epigenetic alterations persisted over time. In fact, almost 30 k cytosines across the genome showed persistent methylation differences over time due to maternal nutrition. Interestingly, the over-representation analysis revealed that some of the genes persistently altered by maternal methionine supplementation are closely related to DNA and histone methylation. To the best of our knowledge, this is one of the first studies showing that methylation changes induced by maternal nutrition in the offspring epigenome can persist over time. Note that alterations in the methylation patterns that persist into adulthood can exert long-term effects and have potentially long-life consequences.

Maternal methionine supplementation persistently altered the methylation of genes closely related to muscle physiology, such as muscle contraction. Interestingly, previous studies in beef and sheep have also shown that maternal nutrition during gestation can alter the expression of genes related to muscle contraction in the offspring^27,28^. Similarly, previous studies in pigs have shown that supplementation during pregnancy with methylation-related micronutrients, such as folate, B6, B12, methionine, choline, and zinc, can promote sex-specific myogenic maturation processes related to growth and muscle metabolism^29^. Maternal methionine supplementation also persistently impacted the cytoskeleton of the muscle cells, and in particular the microtubules. Microtubules play important roles in different cellular processes, such as intracellular trafficking, cell division, and maintenance of cellular architecture, including shape, polarity, and organelle positioning^30^. There is growing evidence that maternal dietary manipulations can alter the cytoskeleton of muscle cells in the offspring. For instance, Max and collaborators have shown that maternal vitamin D deficiency in rats alters the expression of genes involved in myogenesis and cytoskeleton organization, including microtubule-associated protein tau, in skeletal muscle of the newborns^31^.

Some of the genes that showed persistent DNA methylation changes are closely related to mitochondrial function, reactive oxygen species (ROS) metabolic process, and redox regulation. These results are in concordance with previous studies that have shown that maternal nutrition during gestation can significantly alter muscle mitochondrial function and ROS homeostasis in the offspring^32–34^. Note that mitochondria are key organelles involved in generating ATP via oxidative phosphorylation, but apart from this main role, mitochondria also have other functions, including apoptosis regulation, calcium homeostasis, and ROS production^35^. ROS are in general considered as toxic species resulting in oxidative stress, pathogenesis, and aging. However, there is growing evidence that ROS are also important signaling molecules regulating physiological processes. Indeed, in skeletal muscle, ROS activate and/or participate in many signaling pathways, promoting complex and diverging effects, ranging from differentiation and adaptation to autophagy and cell death^36^. Similarly, recent research has shown that mitochondria can modulate the fate of the myogenic stem cell population, known as satellite cells, either to maintain quiescence, activate, self-renew, or differentiate^37^. This is particularly important given that environmental insults, such as maternal nutrition, could alter mitochondrial function and ROS homeostasis, which in turns could alter the fate of muscle satellite cells. In livestock, these alterations may have long-term consequences, affecting muscle growth, muscle composition, and meat quality.

Autophagy was one of the biological processes most impacted by maternal methionine supplementation. Autophagy is a highly conserved homeostatic process in charge of degrading cytoplasmic components, including damaged organelles and toxic protein aggregates^38^. It is well-documented that autophagy is important to preserve skeletal muscle mass and to maintain myofiber integrity^39^. Indeed, alteration of autophagy in the skeletal muscle has been associated with accumulation of abnormal mitochondria, sarcoplasmic reticulum distension, disorganization of sarcomere, and formation of aberrant concentric membranous structures^39^. Notably, our findings agree with previous studies that have reported that either maternal under- or over-nutrition during gestation alters autophagy pathways in both skeletal and cardiac muscle of the offspring^40,41^.

Maternal methionine supplementation also persistently altered the methylation of genes directly implicated in the PIK3 pathway. Phosphoinositide 3-kinases (PI3Ks) are implicated in receptor-stimulated signaling and play a major role in cell metabolism, growth, proliferation, and survival^42^. In muscle, the activation of the PI3K pathway induces skeletal muscle hypertrophy, defined as an increase in skeletal muscle mass^43^. Interestingly, rats with intrauterine growth retardation due to a maternal low-protein diet showed an alteration in the PI3K pathway in skeletal muscle^44^. Similarly, maternal obesity and over-nutrition impaired the PI3K pathway in fetal skeletal muscle in a sheep^45^. Overall, our findings suggest that maternal methionine supplementation might alter PI3K related signaling activities in the skeletal muscle. The ramifications of these alterations, in terms of postnatal muscle growth and muscle composition, warrant future investigation.

## Conclusions

Our study has shown that maternal methionine supplementation during periconception and first trimester of gestation induced persistent DNA methylation changes in the muscle of beef calves. Most genes impacted by persistent DNA methylation changes were related to muscle contraction, mitochondrial function, ROS homeostasis, autophagy, and PIK3 pathway, among other functions. Overall, our study provided additional evidence that maternal nutrition can alter the epigenome of the fetus, and some of these epigenetic changes persisted over time (Supplementary Information).

## Supplementary Information

Supplementary Information 1.

Supplementary Information 2.

Supplementary Information 3.

Supplementary Information 4.

## Data Availability

Bisulfite-Seq data are available on NCBI GEO with accession numbers GSE117194 and GSE218011.
